# Prevention of hospital-acquired bloodstream infections through chlorhexidine gluconate-impregnated washcloth bathing in intensive care units: a systematic review and meta-analysis of randomised crossover trials

**DOI:** 10.2807/1560-7917.ES.2016.21.46.30400

**Published:** 2016-11-17

**Authors:** Elsa Afonso, Koen Blot, Stijn Blot

**Affiliations:** 1 Neonatal Intensive Care Unit, Cambridge University Hospital, Cambridge, United Kingdom; 2 These authors contributed equally to the manuscript; 3 Faculty of Medicine and Health Science, Ghent University, Ghent, Belgium; 4 Department of General Internal Medicine, Faculty of Medicine and Health Science, Ghent University, Ghent, Belgium; 5 Burns Trauma and Critical Care Research Centre, The University of Queensland, Brisbane, Australia

**Keywords:** healthcare-associated infections, infection control, Intensive Care, hygiene, policy, prevention

## Abstract

We assessed the impact of 2% daily patient bathing with chlorhexidine gluconate (CHG) washcloths on the incidence of hospital-acquired (HA) and central line-associated (CLA) bloodstream infections (BSI) in intensive care units (ICUs). We searched randomised studies in Medline, EMBASE, Cochrane Library (CENTRAL) and Web of Science databases up to April 2015. Primary outcomes were total HABSI, central line, and non-central line-associated BSI rates per patient-days. Secondary outcomes included Gram-negative and Gram-positive BSI rates and adverse events. Four randomised crossover trials involved 25 ICUs and 22,850 patients. Meta-analysis identified a total HABSI rate reduction (odds ratio (OR): 0.74; 95% confidence interval (CI): 0.60–0.90; p = 0.002) with moderate heterogeneity (I^2^ = 36%). Subgroup analysis identified significantly stronger rate reductions (p = 0.01) for CLABSI (OR: 0.50; 95% CI: 0.35–0.71; p < 0.001) than other HABSI (OR: 0.82; 95% CI: 0.70–0.97; p = 0.02) with low heterogeneity (I^2^ = 0%). This effect was evident in the Gram-positive subgroup (OR: 0.55; 95% CI: 0.31–0.99; p = 0.05), but became non-significant after removal of a high-risk-of-bias study. Sensitivity analysis revealed that the intervention effect remained significant for total and central line-associated HABSI. We suggest that use of CHG washcloths prevents HABSI and CLABSI in ICUs, possibly due to the reduction in Gram-positive skin commensals.

## Introduction

Hospital-acquired bloodstream infections (HABSI) and the subgroup of central line-associated bloodstream infections (CLABSI) are associated with substantial morbidity, mortality, and healthcare costs in adults and children [1-5], with higher infection rates among hospitalised children [6]. Data from the EPIC II study have shown that of all nosocomial infections in the intensive care unit (ICU), 15% were bloodstream infections (BSI), with CLABSI accounting for 4.7% [7,8]. Due to the substantial impact on patient outcomes and their preventable nature, reduction of HABSI is the emphasis of several patient safety initiatives [9-11].

CLABSI results from catheter tip contamination by commensal skin flora at time of device insertion and later from microorganisms migrating from skin to the catheter tip or lumen [12]. The risk of CLABSI can be reduced by antiseptic skin preparation immediately before catheter insertion and by maintaining asepsis at insertion site and catheter access points [13]. As a substantial proportion of primary BSI originate from vascular access devices, these infections also decrease following preventive interventions targeting CLABSI [14].

Chlorhexidine gluconate (CHG) has broad antimicrobial action, prolonged residual effect, and is the agent of choice for skin disinfection before catheter insertion [13,15,16]. CHG can also be used in basic hygienic care as a liquid bathing agent or as pre-packaged CHG-impregnated washcloths [17].

A substantial number of studies investigated the value of CHG washcloth patient bathing. Three recent systematic reviews summarised the available evidence concerning colonisation and infection rates [18-20]. Low-quality, non-randomised studies demonstrated mixed effects for prevention of BSI. The effect of CHG-impregnated washcloths on hard outcomes such as rates of HABSI and CLABSI in both adult and paediatric ICU patients remains unclear. We performed a systematic review and meta-analysis of randomised-controlled trials to assess the impact of daily care with CHG washcloths on rates of total HABSI and CLABSI in adult and paediatric ICU patients. Subgroup analysis identified the impact on Gram-positive and Gram-negative microorganisms.

## Methods

### Search strategy

**Figure 1:**
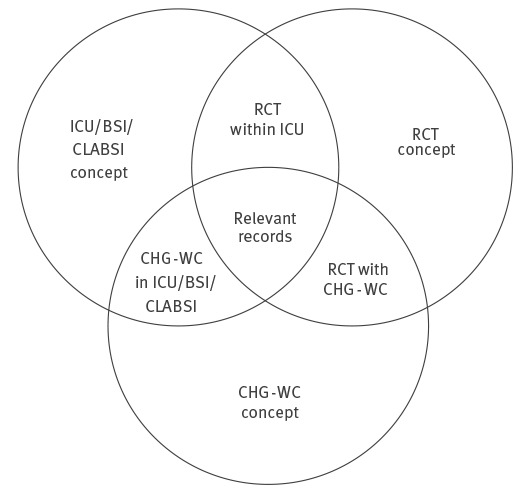
Study selection according to online databases

BSI: bloodstream infection; CHG-WC: chlorhexidine gluconate washcloth; CLABSI: central line-associated bloodstream infection; ICU: intensive care unit; RCT: randomised controlled trial.

The Medline, EMBASE, Cochrane Library and Web of Science databases were systematically searched using combinations of the key terms ‘chlorhexidine’, ‘chlorhexidine impregnated washcloths’, ‘neonatal’, ‘paediatric’ ‘intensive care unit’, ‘bloodstream infection’, ‘catheter related infection’ and ‘randomised controlled trial’ (Box 1-2, Figure 1). The search strategy included publications until end of April 2015. No predefined review protocol was registered.

Box 1 **Systematic Review Protocol**

Inclusion criteria

Randomised controlled trials
Adult ICU population
Paediatric ICU population
Neonatal ICU population
Intervention arm including patient bathing with CHG washcloths
Control arm including other standard bathing procedures (not with CHG or other antiseptic)
Records investigating impact of intervention in HABSI and CLABSI
Full text available

Exclusion criteria

Descriptive studies
Before-and-after design
Evidence of confounders such as other interventions implemented at the same time as CHG washcloth bathing (i.e. care bundles)
Comparative studies
Reviews, systematic reviews and meta-analysis
Studies that did not use CHG in the form of washcloths

CHG: chlorhexidine gluconate; CLABSI: central line-associated bloodstream infection; HABSI: hospital-acquired bloodstream infection; ICU: intensive care unit.

Box 2 **Search terms used for study selection**

1. MEDLINE search (181 titles found, 7 without accessible full text, 159 excluded, 18 duplicates, 4 eligible studies)

Chlorhexidine impregnated washcloths AND catheter related bloodstream infection
Chlorhexidine impregnated washcloths AND bloodstream infection
Chlorhexidine[MeSH Terms] AND infection transmission[MeSH Terms] AND care units, intensive[MeSH Terms]
Chlorhexidine[MeSH Terms] AND bath[MeSH Terms] AND pediatric intensive care units[MeSH Terms]
Chlorhexidine[MeSH Terms] AND bath[MeSH Terms] AND intensive care unit[MeSH Terms]
Chlorhexidine[MeSH Terms] AND care, neonatal intensive[MeSH Terms]
Chlorhexidine[MeSH Terms] AND care unit, intensive[MeSH Terms] AND catheter related infection[MeSH Terms]
Chlorhexidine wash[MeSH Terms] AND BSI
Chlorhexidine impregnated AND CLABSI
Chlorhexidine impregnated AND BSI
Chlorhexidine impregnated AND Pediatric
Chlorhexidine impregnated AND Neonatal
Randomized controlled trial[Publication Type]) AND ICU AND Chlorhexidine
Randomized controlled trial[Publication Type] AND intensive care unit) AND chlorhexidine impregnated
Chlorhexidine[MeSH Major Topic] AND randomized controlled trial[Publication Type] AND ICU

2. EMBASE search (14 titles found, 12 excluded, 2 duplicates)

Bath/ and *chlorhexidine gluconate/ and intensive care unit/
Chlorhexidine washcloths and intensive care unit).af
Chlorhexidine washcloths and neonatal).af
Randomized controlled trial.pt. and chlorhexidine washcloths.af
  3 Web of Science search (81 studies found, 78 excluded, 3 duplicates)
TS=(chlorhexidine AND wash*) AND TS=(intensive care unit)
TS=(chlorhexidine AND wash*) AND TS=(pediatric)
TS=(chlorhexidine AND wash*) AND TS=(neonatal intensive care)
TS=(chlorhexidine AND wash*) AND TS=(BSI)
TS=(chlorhexidine AND wash*) AND TS=(CLABSI)

4. Cochrane Library search (15 titles found, 7 excluded, 8 duplicates)

'Randomized* in Publication Type AND chlorhexidine wash* AND "intensive care unit" NOT "oral"NOT "hand" in Trials'
'Randomized* in Publication Type and chlorhexidine bath* and Intensive Care Unit in Trials'
'Randomized* in Publication Type and chlorhexidine bath* and "intensive care unit" and "neonatal" in Trials'
'Randomized* in Publication Type and chlorhexidine bath* and "intensive care unit" and "BSI" in Trials'
'Randomized* in Publication Type and chlorhexidine washcloth and "intensive care unit" in Trials'

BSI: bloodstream infection; CLABSI: central line-associated bloodstream infection; HABSI: hospital-acquired bloodstream infection; ICU: intensive care unit.

### Study selection

Eligible studies included randomised trials done in adult, paediatric and neonatal ICUs that compared the impact of daily bathing with CHG washcloths with that of non-antiseptic impregnated washcloths or other standard bathing procedures on HABSI rates. Languages were restricted to English, French, Dutch and Portuguese. The primary outcome measure was number of HABSIs per patient-days. One reviewer performed study selection and consensus was achieved between two reviewers. Search results were screened by title and abstract. Selected papers underwent a full-text assessment and eligibility issues were resolved between reviewers (EA, KB, SB).

### Data extraction and quality assessment

Extracted data included study setting, design and sample size, implemented interventions, definitions and primary outcome data on rates of CLABSI and HABSI per patient-days in the treatment and control groups from the intention-to-treat populations. Data were manually calculated when necessary. Secondary outcome measures included Gram-positive and Gram-negative aetiology, study-related adverse events and number of catheter-days and patients. When available, the protocols were examined for discrepancies between original study objectives and the published data. Two independent reviewers performed data extraction and independently assessed the methodological quality of included studies using the Cochrane risk-of-bias assessment tool (EA, KB) [21].

### Statistical analysis

A random-effects meta-analysis using the inverse variance method obtained odds ratios (OR) and 95% confidence intervals (CI) for total HABSI rates per 1,000 patient-days. A random-effects model was chosen to encompass clinical heterogeneity in baseline standards of care between ICUs. Heterogeneity was predefined and assessed through the I^2^ test (I^2^ ≤ 25% for low, 25% < I^2^ < 50% for moderate and I^2^ ≥ 50% for high). Predefined subgroup analysis and meta-regression were performed on HABSI subtype (CLABSI and non-central line HABSI) and pathogen subtype (Gram-negative and Gram-positive). Sensitivity analysis assessed the impact of varying incidence rate denominator data (number of catheter-days and patients) and the removal of studies with a high risk of bias. Assessment of publication bias by funnel plot was planned when considered meaningful (i.e. at least 10 studies included). Review Manager version 5.2.0 was used for meta-analysis models and Comprehensive Meta Analysis version 2.0 was used to perform meta-regression. A p value ≤ 0.05 was considered statistically significant.

## Results

**Figure 2:**
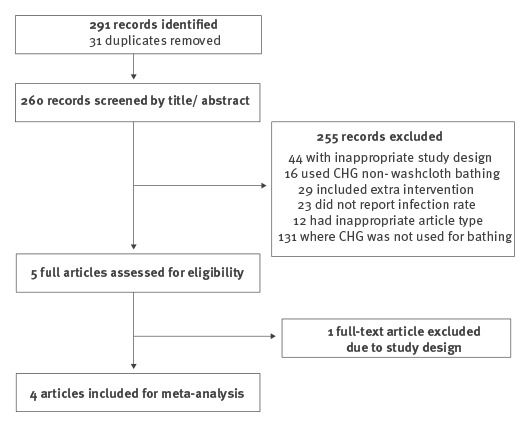
Summary of literature search and study selection (n = 291)

The search strategy yielded 291 records. Following title, abstract and full-text assessment, four papers were included for meta-analysis (Figure 2) [22-25], and one study was excluded because it had an inappropriate non-randomised study design. The included studies were non-blinded cluster-randomised crossover trials involving, together, 22,850 patients from 15 adult and 10 paediatric ICUs (Table 1). The treatment group included daily patient bathing with 2% CHG washcloths. Control groups applied daily bathing with non-antiseptic impregnated washcloths or other non-medicated standard bathing procedures in ICUs with comparable baseline infection rates.

CHG: chlorhexidine gluconate.

**Table 1:** Summary of included studies (n = 4)

Study	Setting	Sample size	Intervention group	Control group	Primary outcome	Secondary outcome	Results
**Climo (2013) [** **22** **]**	9 ICU and bone marrow transplant units	7,727 patients	CHG-WC daily bathing	Daily bath with non-medicated washcloths	Primary, secondary, and central line-associated BSI	Primary HABSI and CLABSI microorganisms	Control: 88 primary HABSI, 43 CLABSI, 34 secondary HABSI for 25,000 patient-days. Intervention: 69 primary HABSI, 21 CLABSI, 29 secondary HABSI for 24,931 patient days.
**Noto (2015) [** **23** **]**	5 adult ICUs (neurological, trauma, surgical, medical, cardiovascular)	9,340 patients	CHG-WC daily bathing	Non-medicated washcloths	Combined primary and secondary HABSI, and CLABSI	Combined primary, secondary HABSI, and CLABSI microorganisms	Control: 113 primary and secondary HABSI, 4 CLABSI for 20,721 patient-days. Intervention: 96 primary and secondary HABSI, 4 CLABSI for 19,202 patient days.
**Bleasdale (2007) [** **24** **]**	1 medical ICU	836 patients	CHG-WC daily bathing	Soap and water bathing	Combined primary HABSI and CLABSI, and secondary HABSI	Combined primary HABSI and CLABSI microorganisms	Control: 21 CLABSI, 1 primary HABSI, 5 secondary HABSI for 2,119 patient days. Intervention: 9 CLABSI, 0 primary HABSI, 5 secondary HABSI for 2,210 patient days.
**Milstone (2013) [** **25** **]**	10 paediatric ICUs	4,947 patients	CHG-WC daily bathing	Either soap and water or non-medicated washcloths	Combined primary and secondary HABSI, and CLABSI	Combined primary HABSI and CLABSI microorganisms	Control: 79 primary and secondary HABSI, 28 CLABSI for 16,024 patient days. Intervention: 53 primary and secondary HABSI, 13 CLABSI for 15,057 patient days.

ICU: intensive care unit; BSI: bloodstream infection; CHG-WC: 2% chlorhexidine gluconate washcloth; CLABSI: central line-associated bloodstream infection; HABSI: hospital-acquired bloodstream infection.

The four included studies compared daily 2% CHG washcloth patient bathing with a control arm applying washcloths not impregnated with CHG: non-medicated washcloths [22,23], soap and water [24] or not further specified non-medicated standard bathing procedures [25]. Climo et al. performed their study in nine medical adult ICUs and bone marrow transplantation units [22] and Bleasdale et al. performed a single-centre study in a medical ICU [24]. Milstone et al. studied the intervention impact in 10 paediatric ICUs [25]. Noto et al. selected five adult ICUs in the same institution (cardiac, trauma, neurological, medical and surgical) [23]. Total HABSI rates in the control arm were comparable between the three multicenter studies (5.5–6.6 HABSI per 1,000 patient-days) [22,23,25], with one study reporting twice larger infection rates (12.2 HABSI per 1,000 patient days) in a single ICU [24]. The rates of CLABSI in the control arm varied per study between 0.19 [23], 1.7 [22,25] and 9.9 CLABSI per 1,000 patient days [24].

All 25 units were randomly assigned to either a treatment or a control group. Duration of study period was 10 weeks [23], 6 months [22,25], and 6 or 7 months in both control and treatment groups [24]. Three of the four studies applied washout phases between control and treatment study periods, lasting two [23,24] or six weeks [25]. The study by Climo et al. did not include a washout phase between intervention and control study periods [22]. Three of the studies reported that nurses received training on how to perform bathing and how to identify adverse events related or unrelated to the treatment [22,24,25]. All four trials were non-blinded to patients, caregivers and staff.

Climo et al., Bleasdale et al. and Noto et al. included all admitted adult patients in the ICUs who agreed to participate except those with adverse skin conditions [22-24]. Eight patients refused to participate in the study by Climo et al. and were not included in the final analysis [22]. Bleasdale et al. excluded three patients who lacked skin integrity, declined participation or developed a skin rash. However, these patients were included in the final intention-to-treat analysis [24]. Milstone et al. used an intention-to-treat approach when selecting paediatric patients for analysis. All children admitted in the paediatric ICU were eligible for this study except those younger than two months, those with a present epidural or lumbar drain, skin disease, burns or CHG allergy or those without parental consent. The intention-to-treat population included all children older than two months with an informed consent to participate, whereas the per-protocol population included all the children who received treatment and were not excluded because of adverse reactions [25]. Finally, Noto et al. stated that all admitted patients were randomised and patients admitted during the washout periods were excluded [23].

Climo et al. and Noto et al. defined primary HABSI as a BSI detected at least 48 hours after admission without an attributable secondary source of infection. Bleasdale et al. used the 1988 definitions from the United States Centers for Disease Control and Prevention (CDC) for nosocomial infections for HABSI and CLABSI [26]. These criteria require catheter cultures to define central line bloodstream infections, as opposed to more recent CLABSI definitions of a HABSI occurring in in a patient with a central line (within 48 hours) with no other clear infectious source. Climo et al. and Noto et al. applied the CDC and National Healthcare Safety Network (NHSN) definitions for CLABSI and HABSI [27]. Noto et al. reported combined primary and secondary HABSI rates. Milstone et al. likewise applied CDC/NHSN definitions, however they defined their bloodstream infections by any single positive blood culture, including for commensal skin microorganisms [25]. The authors justified the adjusted definition criteria by stating that morbidity from bacteraemia is significant in critically ill children. Microorganisms identified as Gram-positive or Gram-negative were isolated from combined primary BSI and CLABSI [22,24,25] or from total HABSI [23].

Three studies evaluated study-related adverse events associated with the use of CHG washcloths [22,24,25], but none reported serious adverse events. Milstone et al. specified the occurrence of skin reactions in 69 (2%) patient admissions, with a greater percentage occurring in the treatment group than in the control group (n = 43 (3%) vs n = 26 (1%); p < 0.0001). Only 28% (12/43) of these reactions were considered to be due to CHG washcloths. Crude incidence of CHG-related adverse events was 1.12 per 1,000 patient-days (95% CI: 0.06–2.02) [25]. One study found a higher overall incidence of skin reactions in the control group (n = 130 (3.4%)) rather than the intervention arm (n = 78 (2%)), with all reactions considered not related to the CHG washcloth bathing intervention [22]. Bleasdale et al. reported three cases of skin reaction in the intervention group, which were likewise not attributed to CHG washcloth use [24]. The study by Noto et al. did not report any adverse events [23]. Only the study by Bleasdale et al. studied the minimum inhibitory concentration (MIC) for chlorhexidine resistance of microorganisms in the control and intervention arms, however neither the data nor significance values were reported.

Risk of bias assessment was performed using the Cochrane Collaboration tool for risk of bias assessment (Table 2) [21]. Besides the inability to blind the intervention to patients and staff and the lack of compliance measurements for interventions or baseline hygienic practices in all studies, the main confounder that introduced a high risk of bias was the simplified definition applied by Milstone et al. for their paediatric population. In that study, only one positive blood culture was required to diagnose a bloodstream infection, including commensal skin microorganisms. In the same vein, none of the included articles reported diagnostic methods of catheter or blood culturing. Other sources of bias included lack of a washout phase and no mention of outcome assessment blinding in the Climo et al. study. Issues that confounded generalisability include higher HABSI rates in the Bleasdale et al. trial and lower HABSI rates and shorter mean length of stay in the Noto trial.

**Table 2:** Cochrane risk-of-bias assessment of included studies (n = 4)

	Climo (2013) [22]	Noto (2015) [23]	Bleasdale (2007) [24]	Milstone (2013) [25]
**Random sequence generation and allocation concealment**	Investigators were unblinded to intervention assignment. No mention of blinding of outcome assessments.	Infection control personnel responsible for adjudicating infection outcomes were blinded to the treatment assignments.	One of three reviewers was blinded to intervention assignment. To avoid bias, infection rates were calculated with a computer algorithm on a data warehouse.	Investigators were unblinded to intervention assignment. Outcome assessors were masked to random allocations.
**Selection bias**	Medium risk	Low risk	Low risk	Low risk
**Blinding of participants and personnel**	Due to the nature of the study, none of the studies could blind intervention to staff or patients.			
**Blinding of outcome assessment**	Investigators were unblinded to intervention assignment. No mention of blinding of outcome assessments.	Infection control personnel responsible for adjudicating infection outcomes were blinded to the treatment assignments.	Two reviewers were unblinded to intervention assignment; a third reviewer was blinded. To avoid bias, infection rates were electronically calculated using a computer algorithm on a data warehouse.	Investigators were unblinded to intervention assignment. Outcome assessors were masked to random allocations.
**Performance bias**	Medium risk	Low risk	Low risk	Low risk
**Incomplete outcome data**	Reported cost-effectiveness outcomes did not coincide with the protocol. Adverse events reported. Chlorhexidine susceptibility testing was reported. No compliance reporting.	Reported primary and secondary outcomes coincided with the protocol. No data on chlorhexidine resistance. No compliance reporting.	Reported primary and secondary outcomes coincided with the protocol. Adverse events reported. Chlorhexidine susceptibility testing was reported. No compliance reporting.	Reported primary and secondary outcomes coincided with the protocol. Adverse events reported. No data on chlorhexidine resistance. No compliance reporting.
**Detection bias**	Low risk	Low risk	Low risk	Low risk
**Selective reporting**	Cost-effectiveness data not mentioned in the study report but mentioned in the study protocol. Only intention-to-treat group reported.	Intention-to-treat and as-treated group analysis provided. Adverse events not reported.	Only an intention-to-treat analysis was performed. Three patients excluded from the CHG bathing procedure were considered part of the intervention arm.	Per protocol and intention-to-treat group analysis provided.
**Attrition bias**	Low risk	Low risk	Low risk	Low risk
**Other sources of bias**	Sage Products supplied the washcloths, technical and educational support, but was not involved in the study design, analysis or manuscript preparation.	Single-centre study with lower baseline HABSI rates and length of stay compared with other included studies.	Single-centre study with higher baseline CLABSI rates compared with other studies.	Different institutions’ ethics committees decided how to obtain informed consent. BSI criteria required only one blood culture for commensal microorganisms.
**Other bias**	Low risk	High risk	Medium risk	High risk

BSI: bloodstream infection; CHG: chlorhexidine gluconate; CLABSI: central line-associated bloodstream infection; HABSI: hospital-acquired bloodstream infection.

**Figure 3:**
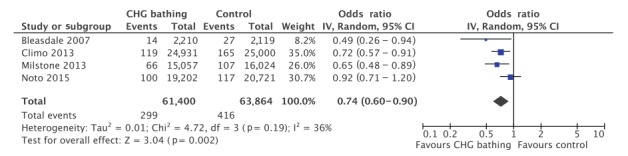
Meta-analysis of the impact of chlorhexidine gluconate washcloth bathing on total rate of hospital-acquired bloodstream infections per patient-days (n = 4 studies)

Meta-analysis was performed on the randomised crossover trials to assess the impact of CHG washcloth bathing. A reduction in the rate of total HABSI was associated with CHG washcloth bathing (OR: 0.74; 95% CI: 0.60–0.90; p = 0.002, Figure 3) with moderate statistical heterogeneity (I^2^ = 36%). One study did not demonstrate a rate reduction of total HABSI [23].

CHG bathing: chlorhexidine-impregnated washcloth bathing; CI: confidence interval; control: bathing with non-impregnated washcloths.

In Climo et al.’s study, CLABSI rate was 53% lower in the CHG washcloth group than in the control group [22]. Bleasdale et al. described a lower CLABSI risk in the CHG washcloth group than in the control group [24]. Milstone et al.’s study on paediatric patients found a decreased incidence of BSI in patients with a central line (p = 0.03). However, CHG washcloth bathing was not associated with a significantly decreased incidence of CLABSI (p = 0.08) [25]. Noto et al. did not report a significant impact of using CHG washcloths on the rates of CLABSI [23].

**Figure 4:**
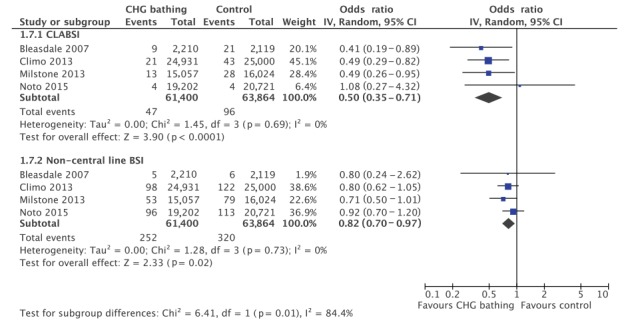
Subgroup analysis of rates of central line-associated bloodstream infection and non-central line-associated hospital-acquired bloodstream infection per patient days (n = 4 studies)

BSI: bloodstream infection; CHG bathing: chlorhexidine-impregnated washcloth bathing; CI: confidence interval; CLABSI: central line-associated bloodstream infection; control: bathing with non-impregnated washcloths.

**Figure 5:**
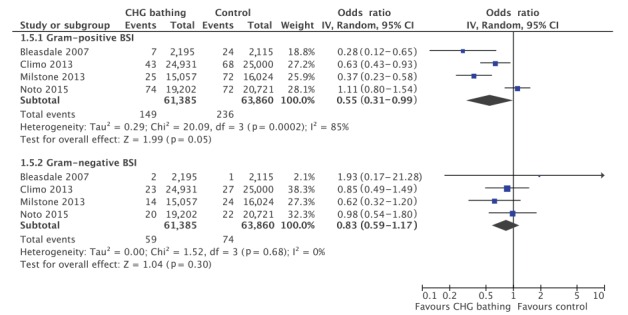
Subgroup analysis of rates of hospital-acquired Gram-positive and Gram-negative bloodstream infections per patient days (n = 4 studies)

BSI: bloodstream infection; CHG bathing: chlorhexidine-impregnated washcloth bathing; CI: confidence interval; control: bathing with non-impregnated washcloths.

Subgroup analysis found a significant reduction in CLABSI (OR = 0.50; 95% CI: 0.35–0.71; p ≤ 0.001, Figure 4) and non-central line-associated HABSI rates per 1,000 patient days (OR = 0.82; 95% CI: 0.70–0.97; p = 0.02). Both subgroups displayed lower heterogeneity compared with the total HABSI rate reduction (I^2^ = 0%), demonstrating that heterogeneity between studies is partially explained by which infectious outcome is being studied. The effect of CHG washcloth bathing was more pronounced for CLABSI prevention and the difference in impact was significant (p = 0.01). Three of the four studies reported the cultured microorganisms for combined primary and central line-associated HABSI, while the Noto study reported data on combined primary and secondary HABSI [25]. Subgroup analysis found a significant decrease in Gram-positive (OR = 0.55; 95% CI: 0.31–0.99; p = 0.05) but not Gram-negative HABSI (OR = 0.83; 95% CI: 0.59–1.17; p = 0.68) (Figure 5). Meta-regression did not identify a significant difference between Gram-positive and Gram-negative subgroups.

Noto et al. reported microorganism data on combined primary and secondary hospital-acquired bloodstream infection [23].

A funnel plot was not created due to the small number of included studies. Sensitivity analysis compared meta-analysis results for varying denominators per HABSI. The intervention effect per number of patients was comparable for total HABSI (OR = 0.73; 95% CI: 0.58–0.91; p = 0.006), CLABSI (OR = 0.50; 95% CI: 0.35–0.71; p = 0.0001) and non-central line HABSI (OR = 0.82; 95% CI: 0.68–0.97; p = 0.02). Three trials demonstrated that the overall effect on CLABSI per catheter-days was similar (OR = 0.52; 95% CI: 0.36–0.74; p = 0.0003) with one study demonstrating a non-significant decrease [25]. The definitions of HABSI in the paediatric population of Milstone et al. required only one blood culture, even in the case of skin commensals. After exclusion of this high-risk-of-bias study, meta-analysis identified a reduction of the total HABSI (OR = 0.76; 95% CI: 0.59–0.99; p = 0.04) and CLABSI rate per patient days (OR = 0.50; 95% CI: 0.33–0.76; p = 0.02), and the rate reduction for non-central line HABSI, Gram-positive and Gram-negative HABSI became non-significant. The difference between CLABSI and non-central line HABSI remained significant after removal of this high-risk-of-bias trial (p = 0.02).

## Discussion

This meta-analysis of four trials, involving 25 ICUs and 22,850 patients, provides evidence that daily patient bathing with CHG washcloths can reduce the incidence of HABSI. This effect appears mainly to be due to a reduction in CLABSI, possibly based on eradication of Gram-positive skin commensals. After removal of a high-risk-of-bias study, the intervention impact in the Gram-positive and non-central line-associated HABSI subgroups became non-significant. No significant adverse skin events were reported as related to CHG washcloth bathing. One study planned a cost-effectiveness analysis per protocol, but did not report this in the final publication [22]. Among the four studies, significant reductions in individual infection rate were demonstrated for total HABSI (n = 3) and for the subgroups of Gram-positive HABSI (n = 3) and CLABSI (n = 3). The subgroup analysis of non-central line HABSI demonstrated rate reductions, however no single study could independently demonstrate significance. Only Noto et al consistently reported non-significant results: in contrast to the other included studies, their CLABSI rate did not change, with broad CIs [23]. A possible explanation could be the infection rate in the control group (0.19 CLABSI per 1,000 patient-days), which was at least 10 times lower compared with other trials [22,25], and the mean length of stay of 2.5 days, approximately half that of the other included studies [22,24,25].

Two previous systematic reviews had found evidence of the preventive effect of CHG washcloth bathing for CLABSI [18] and HABSI [20]. Another could not conclude that CHG washcloth bathing could reduce BSI rates [19]. However, the majority of included studies were low-quality non-randomised before–after studies, did not focus solely on ICU patients, and applied different CHG bathing interventions. In contrast, this review focused on non-rinse CHG washcloth bathing to prevent HABSI in ICUs, including pediatric ICUs.

Strengths of this meta-analysis comprise the comprehensive search strategy, inclusion of high-quality randomised crossover trials, risk-of-bias assessment, random-effects meta-analysis with subgroup analysis of HABSI and pathogen subtypes, low statistical heterogeneity in the HABSI types subgroup analysis and sensitivity analysis of high-risk-of-bias studies and denominator data. Limitations include non-blinding to the intervention, partially compensated by the crossover design, lack of compliance measurements and lack of reporting of baseline hygienic practices. Since all four included trials were carried out in critically ill patients with a high level of dependency on staff, patient self-reporting of compliance and tolerance was not performed. One trial did not report blinding of outcome assessment and lacked a washout period [22] and another was single-centre, even though it included two geographically distinct units that permitted a randomised crossover design [24].

Clinical and methodological heterogeneity stemmed from differing infection rates, varying methods of reporting HABSI types and definition criteria of HABSI in the paediatric study. Different baseline standards of care leave more or less room for improvement and HABSI prevention, which can influence the perceived effect of the CHG washcloth intervention. The Bleasdale study had higher rates of HABSI in the control arm and the Climo study higher rates of CLABSI, compared with other hospital settings. This could have produced interpretation and applicability bias in that a situation with more room for improvement in healthcare quality may predispose the infection rate reduction to be stronger [24]. Nevertheless, in the subgroup analysis of central line and non-central line HABSI, the heterogeneity between studies decreased (I^2^ = 0%), indicating that the intervention effect was related to a proportional decrease in HABSI of central line or non-central line origin. An important source of methodological heterogeneity was the Milstone study due to their definition of bacteraemia as one, instead of two, positive blood culture of commensal skin organisms [25]. According to the current evidence, commensal Gram-positive bacteria cause a large proportion of BSI in children; however, they frequently contaminate blood cultures [28,29]. This change in HABSI definition means that the observed intervention effect may represent a false reduction in the yield of contaminated blood cultures that was due to a decrease in commensal skin flora and not to a reduction in bloodstream infections. After removal of this high-risk-of-bias study, a significant reduction was still maintained for total HABSI rates and particularly for the CLABSI subgroup.

The main purpose of patient safety strategies should be to improve quality of care by reducing the clinical and economic burden of healthcare-associated infections. Studies performed in the pre-surgical context have proven the cost-effectiveness of CHG washcloths for preventing surgical site infection [30]; it is unknown if this could be replicated in the ICU context. Studies have hypothesised that CHG washcloth bathing is potentially cost-effective through prevention of CLABSI and that nurses preferred this method over non-washcloth bathing [31-33].

An important concern raised regarding application of antiseptics is the potential selection of antiseptic-resistant pathogens, which should be monitored when introducing universal decolonisation strategies [34]. Only one study measured CHG MIC values between treatment arms, but reflected that the overall increase in resistance in the chlorhexidine group could represent a reduction in isolates that are inhibited by very low CHG concentrations [23].

## Conclusion

This meta-analysis provides evidence that the use of CHG washcloths prevents HABSI in ICUs. The impact of CHG washcloth bathing appeared to be primarily due to its prevention of CLABSI. This effect was beneficial and comparable for CLABSI in all four studies. The reduction was possibly due to the reduction of commensal Gram-positive skin microorganisms. However, since the rate reduction was primarily due to Gram-positive bacteria, the possibility still remains that the intervention effect is partially explained by a reduction in blood culture contamination. Hospitals with high baseline hygienic standards of care and lower CLABSI rates may benefit less from CHG washcloth bathing; rather, the intervention can work as a ‘safety net’ when basic hygienic preventive measures are breached. Further research should apply separate classifications of primary, secondary and central line-associated HABSI types, should report catheter cultures to diagnose bloodstream infections to increase certainty and lower the risk of bias due to improper attribution of blood culture contaminants, should report baseline hygienic standard of care practices and should attempt to measure compliance with the daily CHG washcloth bathing intervention. A cost-effectiveness analysis can assess the added benefit of CHG washcloth bathing, taking into account differing standards of care.
